# Tracing Cattle Breeds with Principal Components Analysis Ancestry Informative SNPs

**DOI:** 10.1371/journal.pone.0018007

**Published:** 2011-04-07

**Authors:** Jamey Lewis, Zafiris Abas, Christos Dadousis, Dimitrios Lykidis, Peristera Paschou, Petros Drineas

**Affiliations:** 1 Department of Computer Science, Rensselaer Polytechnic Institute, Troy, New York, United States of America; 2 Department of Agricultural Development, Democritus University of Thrace, Orestiada, Greece; 3 Department of Molecular Biology and Genetics, Democritus University of Thrace, Alexandroupoli, Greece; University of Utah, United States of America

## Abstract

The recent release of the Bovine HapMap dataset represents the most detailed survey of bovine genetic diversity to date, providing an important resource for the design and development of livestock production. We studied this dataset, comprising more than 30,000 Single Nucleotide Polymorphisms (SNPs) for 19 breeds (13 taurine, three zebu, and three hybrid breeds), seeking to identify small panels of genetic markers that can be used to trace the breed of unknown cattle samples. Taking advantage of the power of Principal Components Analysis and algorithms that we have recently described for the selection of Ancestry Informative Markers from genomewide datasets, we present a decision-tree which can be used to accurately infer the origin of individual cattle. In doing so, we present a thorough examination of population genetic structure in modern bovine breeds. Performing extensive cross-validation experiments, we demonstrate that 250-500 carefully selected SNPs suffice in order to achieve close to 100% prediction accuracy of individual ancestry, when this particular set of 19 breeds is considered. Our methods, coupled with the dense genotypic data that is becoming increasingly available, have the potential to become a valuable tool and have considerable impact in worldwide livestock production. They can be used to inform the design of studies of the genetic basis of economically important traits in cattle, as well as breeding programs and efforts to conserve biodiversity. Furthermore, the SNPs that we have identified can provide a reliable solution for the traceability of breed-specific branded products.

## Introduction

The domestic cow (*Bos taurus*) represents one of the most economically and culturally important species of the planet, providing a significant source of nutrition for the entire human population. More than 800 cattle breeds have been selected by humans for different traits, such as milk yield, meat quality, draft ability, and tolerance or resistance to disease and pests, as well as for social and religious reasons. Modern cattle are thought to have originated from two domestication events of aurochs (*B. primigenius*) in southwest Asia and south Asia resulting to the humpless taurine (*B. taurus*) and the humped zebu (*B. indicus*) groups respectively [1], [2], [3]. Initial domestication is thought to have occurred sometime in the Neolithic (8,000-10,000 years ago) and the subsequent spread of cattle throughout the world is intertwined with human migrations and trade [4]. Today, more than 1.5 billion cattle exist, a number which is expected to grow to 2.6 billion by 2050, according to the Food and Agriculture Organization (F.A.O.) [5].

The study of the bovine genome and the genetic diversity found within and across cattle breeds can provide important insights into mammalian biology and evolution, as well as on the impact of domestication on the species. Population genetic studies of cattle can also have significant economic impact, opening novel opportunities for cattle breeding through genomic selection. Furthermore, they can provide important resources for the conservation of valuable intra-species genetic diversity, which is currently threatened by breed substitution, indiscriminate crossbreeding, and even the absence of breeding programs. Early studies of cattle genomic diversity mainly focused on the analysis of sparse data from microsatellite markers [6], [7], [8], [2], [9], [10], [11]. More recently, studies that evaluated bovine population structure have used Single Nucleotide Polymorphisms (SNPs) [12], [13], [14], [15]; however they focused on a small number of markers. The advent of modern high-throughput technologies is starting to produce genomewide data for thousands of markers across the bovine genome [16], [17], [18], [19], [20]. Undoubtedly, as was the case for studies of human population genomic variation, studies of cattle population genetic structure and variation will be catalyzed by the recent publication of two draft assemblies of the bovine genome [21], [22].

The recent release of data by the Bovine HapMap Consortium [19] represents the most detailed survey of bovine genetic diversity to date. The group reported analyses from the study of 501 animals from 19 worldwide taurine (*B. taurus*), zebu (*B. indicus*), and hybrid breeds (taurine-zebu hybrids), as well as two outgroup species (Anoa and Water Buffalo). This sample was assayed for more than 30,000 SNPs covering the entire bovine genome. The study supported the fact that cattle have undergone a rapid decrease in effective population size from a very large ancestral population, possibly due to domestication and artificial selection [19]. Based on this data, an analysis of the haplotype block structure of the bovine genome revealed two major bottlenecks in bovine history [23]. The first bottleneck is associated with the initiation of cattle domestication. The second bottleneck is much more recent and much more severe and is associated with the intensification of population isolation over the last 700 years. The data also revealed the fact that genomewide data can be used to broadly cluster cattle breeds into groups (zebu, taurine, or hybrid breeds). Thus far, no study has attempted the selection of a small set of markers that can effectively be used for inference of population structure and ancestry (Ancestry Informative Markers – AIMs) from this dataset. Such sets of AIMs could be used to correctly and cost-effectively assign unknown individuals to specific breeds.

The Bovine HapMap dataset [19] provides a unique opportunity to study the genetic structure of diverse cattle populations, using information from the entire bovine genome. As the volume of genotypic data for population genetic studies rapidly increases, a linear dimensionality reduction technique (Principal Components Analysis – PCA) has emerged as a powerful tool for extracting the structure in genomewide datasets [24], [25], [26], [27], [28] offering advantages over the use of computationally intensive model-based algorithms such as those implemented in STRUCTURE [24]. At the same time, the identification of AIMs from genomewide datasets is a topic that has attracted considerable attention due to the value of such markers in diverse areas, ranging from forensics and to population genetics to conservation genetics. Different metrics have been proposed in order to select such markers. Most of them, such as 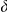 (the absolute difference in allele frequency between two ancestral populations) or Wright's 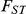 rely on the maximization of allele frequency differences between pre-defined populations [29], [30], [31], [32], [33], [34], [35]. A closely correlated measure, Informativeness for assignment (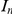) as defined by Rosenberg et al. [36] computes a mutual information based metric on allele frequencies, again demanding the analysis of pre-defined populations. Based on PCA, we have previously described an unsupervised algorithm that can be used to select small subsets of genetic markers (SNPs) that correlate well with population structure, as captured by PCA (PCA Informative Markers – PCAIMs) [26], [37]. Our method can be used to detect SNPs that differentiate individuals from different populations, without any prior knowledge or hypotheses about the data, and without the need to artificially assign individuals to clusters. The efficiency of these PCA-based algorithms has been demonstrated in genomewide studies of human population genetic structure [26], [37].

Leveraging the power of PCA, we set out to investigate whether individual cattle samples can be assigned to specific breeds using only genotype data. Our first goal was the accurate classification of individual cattle from the Bovine HapMap dataset [19] to their ancestral populations using all available genotype data (30,000 SNPs). Our second goal was to further explore the accuracy of such classification tasks while using only small panels of AIMs. Towards that end, we chose to split the main task of classifying samples to breeds into hierarchical levels, splitting the entire cattle population into nested groups which are organized as a decision tree. Applying our SNP selection algorithms [26], [37], we chose small subsets of SNPs that almost perfectly reproduce population structure as identified by PCA and can be used to accurately assign individuals to one of 19 breeds.

## Results

### Breaking down the structure of bovine populations

**Figure 1:**
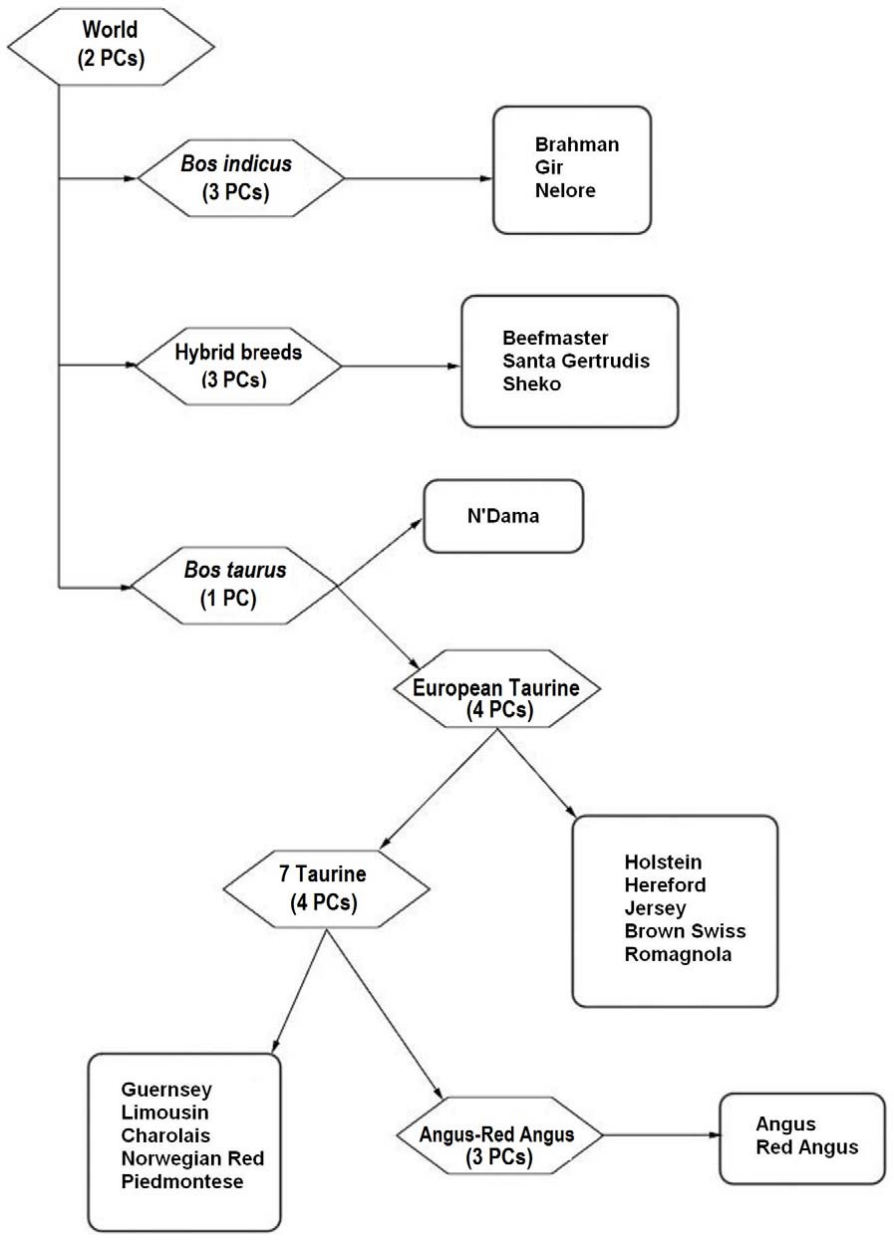
Decision tree for bovine classification. The decision tree for individual assignment to a particular breed (or group of breeds). For each diamond-shaped node we propose (small) panels of AIMs that may be used to assign an individual to one of its children nodes. The rows of square-shaped nodes indicate breed (or groups of breeds) of origin that we can separate. For example, using the panel that we proposed at the World node, we can assign a sample to either *B. indicus*, or *B. taurus*, or hybrid breeds.

**Figure 2:**
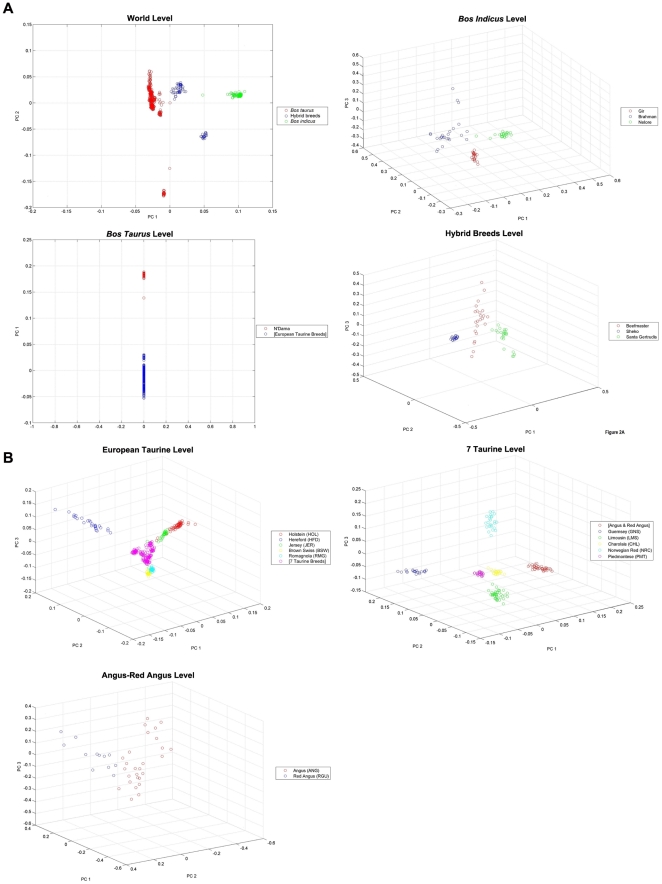
PCA plots. PCA plots at various levels of the decision tree of Figure 1. *(A)* Top left: PCA plot at the World node. Top right: PCA plot at the *B. indicus* node. Bottom left: PCA plot at the *B. taurus* node. Bottom right: PCA plot at the Hybrids node. *(B)* Top left: PCA plot at the European Taurine node. Top right: PCA plot at the seven-taurine node. Bottom left: PCA plot at the Angus-Red Angus node.

We divided the main task of classifying individuals by breed into a sequence of hierarchical levels organized into a decision tree (see Figure 1). The nested groups were chosen by determining clusters of breeds which can be easily differentiated along the significant principal components using all available SNPs (and a standard 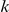-means clustering approach) and then recursively looking at the principal components of each subgroup in the same manner (see Figure 2).

Of the 501 individual cattle and 34,884 genotyped SNPs in the Bovine HapMap dataset [19], 497 cattle and 30,501 SNPs were used in our analysis. We did not analyze the Anoa and Water Buffalo populations, comprising four animals in total, which were used as outgroups in the original study and had more than 50% missing entries in their genotypes. We also removed from consideration approximately 4,000 SNPs due to a high percentage of missing entries (over 10%). A total of 19 worldwide breeds were included in our study, comprising of three zebu breeds, 13 taurine breeds, and three hybrid breeds. At the highest level in the decision tree, individual cattle are broadly classified into one of three groups: *B. taurus*, *B. indicus*, or hybrid breeds. Moving down the decision tree, individuals are more specifically classified into sub-groups until they are finally assigned to an individual breed. The number of nodes in the decision tree depends on the complexity of the initial group and the successive subgroups. Thus, at the *B. indicus* node (see Figure 1), we are differentiating between three cattle populations, namely the Brahman, Gir, and Nelore populations. On the other hand, the *B. taurus* group includes 13 breeds, and as many as four additional levels are needed in order to fully classify an individual into a specific breed.

For instance, in order to classify an “unknown” Red Angus individual using the decision tree of Figure 1 (see also Tables 1 and 2), we first determine whether the individual is part of the *B. taurus* group. We then decide whether the individual belongs to the African N'Dama population or to the European taurine breeds. We then proceed to differentiate between the Holstein, Hereford, Jersey, Brown Swiss, and Romagnola populations and a group that we designate as “seven-taurine-breeds.” The seven-taurine-breeds level of the hierarchy allows us to differentiate further between the Guernsey, Limousine, Charolais, Norwegian Red, and Piedmontese populations, and the Angus-Red Angus group. Finally, we distinguish between the Angus and Red Angus breeds.

**Table 1:** Significant PCs and panel sizes.

Decision Tree node	sign. PCs	Panel P1	Panel P2	Panel P3
		# of SNPs	# of SNPs	# of SNPs
World	2	100	200	300
*Bos taurus*	1	10	20	30
*Bos indicus*	3	50	100	150
Hybrid breeds	3	25	50	75
European taurine breeds	4	100	200	300
7 taurine breeds	4	150	300	450
ANG-RGU	3	25	50	75

Number of significant principal components and AIM panel sizes at each node of the decision tree depicted in Figure 1. Notice that panel P2 contains twice the number of AIMs in panel P1 and panel P3 contains three times the number of SNPs in panel P1.

**Table 2:** Classifying Angus samples.

Angus	Panel 1		Panel 2		Panel 3	
*Decision Tree Nodes*	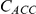	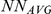	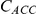	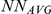	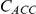	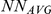
World 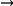 *Bos taurus*	27/27	5.00	27/27	5.00	27/27	5.00
*Bos taurus* 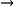 European taurine breeds	27/27	5.00	27/27	5.00	27/27	5.00
European taurine breeds 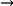 7 taurine breeds	27/27	5.00	27/27	5.00	27/27	5.00
7 taurine breeds 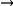 Angus-Red Angus	24/27	4.44	27/27	4.78	26/27	4.85
Angus-Red Angus 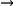 Angus	21/27	3.89	26/27	4.67	25/27	4.3

Predicting the breed of individuals in the Angus (ANG) bovine population using our PCAIM SNP panels P1, P2, and P3. A total of 27 ANG individuals were available in the Bovine HapMap dataset. The 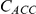 columns correspond to classification accuracy, expressed as the fraction of individuals that were assigned to the correct breed at the respective node of the decision tree, and 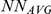 indicates the average number of correct neighbors at the same node of the decision tree. For example, at the seven-taurine-breeds node of the decision tree, 24 out of the 27 Angus samples were (correctly) predicted to be of Angus-Red Angus origin using Panel 1; at the same node, the ANG individuals had – on average – 4.44 neighbors from within the Angus breed.

### Breed inference using the full dataset, five-nearest-neighbors classification, and our decision tree

Our primary goal is the identification of small panels of AIMs that achieve accurate assignment of individuals to breeds, using the reported ancestral breeds in the Bovine HapMap dataset [19] as reference. However, as a first step, we ran a complete leave-one-out crossvalidation experiment using all approx. 30,000 available SNPs in order to assess ancestry inference using the full dataset. Classification was performed by looking at the nearest neighbors of an individual in the space spanned by the significant principal components of the genotype data (see Methods for details). We chose to look at the five nearest neighbors (5-NN classification algorithm) and we assigned an individual to a particular breed if at least three of its five nearest neighbors were from that breed. We defined the classification accuracy to be the percentage of individuals whose predicted breed of ancestry matched the reported reference breed. We also defined a metric focusing on the average number of “correctly predicted” nearest neighbors, i.e., the average number of nearest neighbors that coincide with the reference breed of each individual.

**Figure 3:**
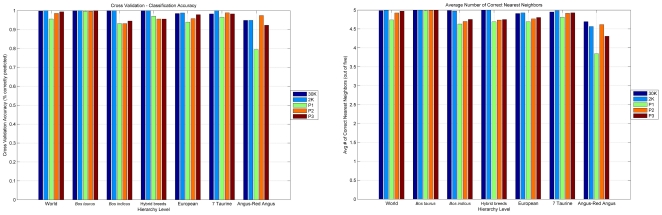
Classification accuracy. Classification accuracy of our complete leave-one-out cross-validation experiment at all nodes of our decision tree. Five different panel sizes are evaluated, with 30K corresponding to all available markers, 2K corresponding to the top 2,000 PCAIMs, and P1, P2, and P3 corresponding to the panel sizes depicted in Table 1. These smaller panels emerged by removing redundant markers from the top 2,000 AIMs. Notice that the top 2,000 markers were selected using only the individuals in the training set of the crossvalidation experiment. *(A)* Classification accuracy results (out of 100%). *(B)* Average number of correctly predicted nearest neighbors (out of five).

Figure 3 summarizes the results of the complete leave-one-out cross-validation experiment for each level of the decision tree in Figure 1. For most nodes in the decision tree the classification accuracy exceeded 98% using the full 30K SNPs dataset (see the dark blue bars in Figure 3A). An exception occurs at the node differentiating between Angus and Red Angus breeds, where the accuracy dropped at 95%. Figure 3B (dark blue bars) illustrates the average number of nearest neighbors (out of a maximum five) that each individual had in the reference breed of origin at each node. This latter plot underlines the power of the proposed method: not only the majority (at least three out of five) of the nearest neighbors of an individual are in the “correct” breed, but in the vast majority of cases (almost) all five neighbors are found in the “correct” breed. The lowest number (4.69 out of five) is again observed in the case of Angus and Red Angus populations. Obviously, even this low number is actually quite close to optimal.

It should be noted that this experiment was also used to determine the number of significant principal components at each node of the decision tree. These numbers were subsequently used for the selection of PCAIMs. See Methods for more details as well as Table 1 (second column).

### Inferring bovine breeds using small panels of AIMs, five-nearest-neighbors classification, and our decision tree

We next focused on selecting small panels of AIMs in order to accurately reproduce the excellent results of breed inference using all 30,000 available SNPs. Towards that end, we evaluated the performance of the AIM selection methods that we previously proposed in [26], [37]. Once more, we ran a full leave-one-out crossvalidation experiment, where we successively treated each of the 497 cattle as a test individual and used the remaining 496 cattle as the training set. AIM panels were selected using only the individuals in the training set. Then the test set subject was classified using our 5-NN algorithm and the decision tree of Figure 1. In that manner, we ensured that the test individual's own data do not impact the selection of AIM panels and thus do not bias the selection of SNPs toward those ideally suited for differentiating and classifying the test individual. This crossvalidation experiment simulates how our algorithm would be applied in practice in order to infer the breed of an unknown individual. As a first step, we selected at each level the top 2,000 PCAIMs, using the number of significant principal components of Table 1. The light blue bars in Figure 3A indicate the performance of these 2,000 SNP panels: they are all roughly as accurate as the full dataset (30K SNPs). Looking at the average number of correctly predicted nearest neighbors (light blue bars in Figure 3B), we observe that even the smallest value exceeds 4.5 out of five, which is a strong indication that our 5-NN approach works well with the selected PCAIM panels. Again, the lowest average number of correctly predicted nearest neighbors is observed at the Angus-Red Angus node (4.56 out of 5).

We have observed in prior work [37] that panels of AIMs selected using PCA scores in genomewide datasets tend to contain large amounts of redundant markers, mainly due to linkage disequilibrium (LD) between densely typed markers. Thus, our next step was the removal of redundant markers via a method that we proposed in [37]; see Methods. We experimented with numerous panel sizes and we chose to report results on three different panels (P1, P2, and P3) for each node in our decision tree. The panel sizes were chosen to maximize classification accuracy with an approximately minimal number of markers and are connected: the number of markers in P2 is equal to twice the number of markers in P1, and the number of markers in P3 is equal to three times the number of markers in P1. The number of markers at each node for each panel is shown in Table 1. Not surprisingly, the number of markers necessary for breed inference is different at the various nodes of the decision tree, reflecting the fact that certain (groups of) breeds are more or less genetically homogeneous. By inspecting Figure 3A and Table 1, we immediately conclude that, within the setting of this experiment, 200 SNPs suffice to classify an individual to one of the three broad species groups (taurine, zebu and hybrid breeds) at the topmost node of our decision tree with an accuracy of 98.6%.

A few interesting observations arise from Figure 3. First, even our smallest panels of AIMs (P1) achieve very high accuracy at most nodes of our decision tree. Not surprisingly, the worst performance happens at the Angus-Red Angus node. In this case, using 25 markers we can achieve 79.4% classification accuracy, which improves to 97.4% using 50 markers. No other panel is associated with a classification accuracy of under 90%. We also observe that, in general, our largest panels (panel P3) perform as well as the top 2,000 PCAIMs before the redundancy removal step. This seems to reinforce the the conjecture that redundancy removal from the top PCAIMs does not significantly affect classification performance, while considerably reducing the number of markers. Finally, we should note that the behavior of our second statistic (average number of correct nearest neighbors) follows a similar pattern with classification accuracy.

We conducted the same experiment using even smaller panel sizes P1 = 10, P2 = 25, and P3 = 50 for all nodes in the hierarchy (Figure S1). The results are, naturally, less accurate at those nodes for which we had previously used more SNPs. However, it is worth noting that, at every node, we were able to successfully assign at least 92% of the studied cattle to the correct breed of origin using panels of 50 PCAIMs (or even fewer in some cases) with on average of 4.5 (out of five) nearest neighbors in the correct population.

In an effort to provide the most comprehensive list of AIMs given the HapMap Bovine reference dataset, we repeated the PCAIMs selection procedure using all available individuals (497 cattle from 19 breeds, see Figures S2 and S3). The list of SNPs needed at each node of our decision tree for accurate (more than 95% classification accuracy at all nodes for our largest panels P3) assignment of individual cattle to one of 19 breeds is presented in the detailed online material that accompanies this paper and is available at http://www.cs.rpi.edu/~drinep/BOVINEPCAIMS/.

## Discussion

The recent release of the Bovine HapMap dataset [19] provides an unprecedented opportunity to study in detail the genomic variation and genetic structure of worldwide cattle breeds, providing an important resource for the design and development of livestock production and filling a void in the study of mammalian evolution [38]. Using this dataset as reference, we have identified small panels of SNPs that can be used to successfully assign unknown cattle samples to one of 19 worldwide breeds. In doing so, we present a thorough examination of population genetic structure in modern bovine breeds. Genotypes from more than 30,000 SNPs were analyzed for 497 individuals [19]. A hierarchical decomposition of the worldwide bovine population was formed, thus enabling the step-wise assignment of individuals to their population group and, ultimately, breed of origin, as well as the sequential selection of subsets of genetic markers that can be used for ancestry and breed inference [26]. Moving through the proposed decision tree investigators have the opportunity to tailor their needs for marker selection according to the desired level of resolution and/or prior information on the origin of the samples under study. The reduction in the number of markers needed is achieved using a redundancy removal algorithm which we have also introduced in prior work [37].

Through this scheme we achieve close to 100% prediction accuracy of individual ancestry when this particular set of 19 breeds is considered, with 250–500 SNPs. To select bovine AIMs we used a PCA-based method that we have previously described [26], [37] (see Methods), which leverages the power of PCA to extract the fundamental axes of variation from a genotypic dataset. Performing a full leave-one-out cross-validation experiment, we showed that in most cases the number of genetic markers needed for ancestry inference can be successfully reduced to less than 1.5% of the original 30,000 SNPs while achieving over 92% accuracy in ancestry prediction. This holds even when closely related breeds are considered. For example, the Angus and Red Angus breeds were largely undistinguished from one another for much of their history, with red animals breeding true amongst the predominantly black Angus cattle populations in coastal England and Scotland. It was only 60 years ago that herds of exclusively red colored Angus cattle were bred separately. Our results confirm the close genetic relatedness between the recently diverged populations of Angus cattle, placing them at the very bottom of our decision tree. However, even for these closely related breeds, a carefully selected panel of approximately 50 SNPs achieves more than 92% differentiation accuracy.

The bovine genome has been shaped by the processes of domestication and artificial selection resulting in dramatic losses of genetic diversity in modern cattle. Studying the Bovine HapMap dataset, Villa-Angulo et al. [23] have shown a persistent decline in effective population size, suggesting two distinctive time points: the time of initial domestication and a time at 100 generations ago, which is associated with intensification of population isolation. The zebu breeds have been shown to be more diverse than the taurine breeds. In agreement with this finding, only two steps are needed in our decision tree in order to classify individuals to one of the three zebu breeds studied, while three to five steps are needed to discern the European taurine breeds. It is also interesting to note that, while continental ancestry is easy to differentiate (i.e., it is easy to differentiate among European, Asian and African breeds), within Europe, taurine breeds do not seem to cluster according to geography. Furthermore, in concordance with previous findings [19], [23], they do not cluster based on their use for dairy of beef products.

The panels that we propose can become important tools in a variety of different settings ranging from comparative genomics to the traceability of bovine products. They might be useful towards studying the evolutionary history, the process of domestication, and the genetic relatedness among modern cattle breeds. They can also be used in the search for phenotype to genotype correlations, especially regarding complex traits, where unidentified population genetic structure can lead to spurious correlations or mask true associations. The sets of markers that we have identified also represent an important resource for the conservation of cattle genetic variation and the design of breeding programs. With genetic diversity in cattle rapidly declining in recent years, genetic tools will undoubtedly prove essential in order to preserve the ability of cattle to respond to changes in the environment, disease challenges, or demand patterns. As extinction of indigenous breeds is accelerated, it will become extremely important to enrich the Bovine HapMap database with information on genetic variation from indigenous animals that have not yet been studied. Given such reference datasets, our approach could be expanded to include additional breeds from around the world, aiding the design of programs to conserve the biodiversity of indigenous breeds.

Importantly, our SNP panels can become a valuable resource for the traceability of bovine products which involves tracking (the ability to follow a product through the supply chain from the farm to the consumer) and tracing (the ability to identify the origins of an item upstream in the supply chain) [15]. Europe has recently seen a trend to promote local and regional food products, leading to the PGI (Protected Geographic Indication) and PDO (Protected Designation of Origin) labels (European Union Regulation (EEU) 2081/92). These labels are meant to support diversity in agricultural production, protect consumers, and protect product names against fraud and imitation [39], [40], [41], [42]. In general, traceability is essential in food safety control, since it facilitates disease control procedures and contributes to consumer confidence in product safety. In many countries, existing tracking systems simply rely on the use of animal tags, tattoos, and computerized barcoded labels. However, over the past ten years, DNA-based systems for traceability of bovine meat and other products have become available and applied commercially (see, for example, http://www.identigen.com/; http://www.pfizeranimalgenetics.com/; http://www.geneseek.com/). The panels of genetic markers that we present, combined with the proposed algorithms, can augment and enhance existing methods, providing accurate and reliable solutions and helping to protect rural communities and regional development.

In conclusion, we have presented a thorough investigation of the genetic structure of 19 worldwide cattle breeds, analyzing the most complete catalogue of bovine genetic diversity to date, the Bovine HapMap dataset [19]. Using methodologies that enable the efficient study of genomewide datasets [26], [37], we have presented a thorough investigation of the genetic structure of 19 worldwide cattle breeds. Our results clearly demonstrate that it is indeed feasible to accurately assign individual cattle to a breed of origin, using in most cases less than a few hundred carefully selected SNPs. Lists of the selected SNPs are available at http://www.cs.rpi.edu/~drinep/BOVINEPCAIMS/. The method that we have used requires no modeling or prior assumptions about the data [26], [37] and has the potential to become an important tool for the study of cattle evolutionary history, as well as studies aiming to uncover the genetic basis of complex and economically important traits in cattle, and conserve biodiversity by informing the design of breeding programs. The sets of SNPs that we propose can also be used to obtain optimum performance based on known characteristics of specific breeds and identify animals for breeding in selection programs. Furthermore, these SNPs can be used to ensure traceability and allow labeling of breed specific branded products. As technologic progress enables the rapid increase of available genotypic data and more breeds encompassing additional aspects of bovine genetic variation are studied in detail, methods like the ones we are proposing here will undoubtedly play a pivotal role in the future of livestock production.

## Methods

### Dataset

We analyzed the Bovine HapMap dataset [19]. Of the 501 individual cattle and 34,884 genotyped SNPs, 497 cattle and 30,501 SNPs were used in our analysis (13 taurine, three zebu, and three hybrid breeds). We removed from our analysis the cattle populations Anoa and Water Buffalo (comprising four cattle in total), as well as all SNPs with more than 10% missing entries (approximately 4,000 SNPs).

### Selecting PCA-Informative Markers (PCAIMs) and removing redundant markers

In order to select AIMs, we leveraged the methods developed in [26], [37]. The method of [26] returns the so-called PCA-score for each SNP, which essentially measures the degree of correlation between a SNP and the significant principal components. The top 2,000 SNPs (those with the highest PCA scores) were subsequently retained. Since the computation of the above scores does not take into account the (potentially high) LD between SNPs, it does result in the selection of many redundant SNPs. In order to remove redundant SNPs, we employ a simple algorithm (presented in [37]) for the Column Subset Selection Problem, which corresponds to the theoretical formulation of the redundancy removal problem. See Methods S1 for additional details on encoding the data and determining the number of significant principal components.

### Five Nearest Neighbors (5-NN) classification algorithm

In order to assign a sample to a population, we used a 5-NN algorithm. Given a target sample, we identify its five nearest neighbors using the standard Euclidean distance in the subspace spanned by the principal components that were deemed significant at the respective node of the decision tree. If at least three of the five nearest neighbors (a majority) belong to the same population, we assign the target sample to that population. We should note that we experimented with different values for the number of nearest neighbors, ranging from three to eleven in increments of two without observing consistent losses or gains in accuracy (data not shown).

## Supporting Information

Methods S1 **Supplementary methods, including details on encoding the data and handling missing entries, determining the number of significant principal components, etc.**
(PDF)

Figure S1 **Classification accuracy with panels of sizes 10, 25, and 50 SNPs**. Classification accuracy of our complete leave-one-out cross-validation experiment at all nodes of our decision tree. Five different panel sizes are evaluated, with 30K corresponding to all available markers, 2K corresponding to the top 2,000 PCAIMs, and P1, P2, and P3 corresponding to panels sizes of 10, 25, and 50 SNPs respectively at all nodes of the decision tree of Figure 1 in the main text. These smaller panels emerged by removing redundant markers from the top 2,000 AIMs. Notice that the top 2,000 markers were selected using only the individuals in the training set of the crossvalidation experiment. *(A)* Classification accuracy results (out of 100%). *(B)* Average number of correctly predicted nearest neighbors (out of five).
(PDF)

Figure S2 **Classification accuracy of proposed panels**. Classification accuracy of our proposed panels at all nodes of our decision tree. Five different panel sizes are evaluated, with 30K corresponding to all available markers, 2K corresponding to the top 2,000 PCAIMs, and P1, P2, and P3 corresponding to the panel sizes depicted in Table 1 of the main text. These smaller panels emerged by removing redundant markers from the top 2,000 AIMs. **Notice that the top 2,000 markers were selected using all 497 samples, without splitting them in training and test sets,** unlike the crossvalidation experiments of Figure 3 (main text). *(A)* Classification accuracy results (out of 100%). *(B)* Average number of correctly predicted nearest neighbors (out of five).
(PDF)

Figure S3 **Classification accuracy of 10, 25, and 50 SNP panels**. Classification accuracy of our “small” panels. Five different panel sizes are evaluated, with 30K corresponding to all available markers, 2K corresponding to the top 2,000 PCAIMs, and P1, P2, and P3 corresponding to panel sizes of 10, 25, and 50 SNPs respectively at all nodes of the decision tree of Figure 1 in the main text. These smaller panels emerged by removing redundant markers from the top 2,000 AIMs. **Notice that the top 2,000 markers were selected using all 497 samples, without splitting them in training and test sets,** unlike the crossvalidation experiment of Figure S1. *(A)* Classification accuracy results (out of 100%). *(B)* Average number of correctly predicted nearest neighbors (out of five).
(PDF)
