# Supplementary material for: Tracing Cattle Breeds with Principal Components Analysis Ancestry Informative SNPs
Source: PLoS One. 2011 Apr 7;6(4):e18007. doi: 10.1371/journal.pone.0018007 (PMC3072384; doi:10.1371/journal.pone.0018007)
Supplement: Methods S1 — Supplementary methods, including details on encoding the data and handling missing entries, determining the number of significant principal components, etc. (PDF) [file pone.0018007.s001.pdf]

Supplementary Material

In order to provide more efficient navigation and access to supplementary material, all the proposed panels of AIMs, as well as additional details of our analysis and numerous PCA plots are available online at the following address (please respect capitalization):

`http://www.cs.rpi.edu/~drinep/BOVINEPCAIMS/`

Supplementary Methods

Encoding the data and handling missing entries

The proportion of missing entries in the Bovine HapMap dataset after our quality control step was approximately 2.45%. It is worth emphasizing that we do not need to fill in missing entries in any manner, since our computations ignore missing entries. We transformed the raw data to numeric values, without any loss of information, in order to apply our linear algebraic methods. More specifically, given a dataset of m samples, and assuming that for each subject n biallelic SNPs have been assayed, we can view the original data as a table consisting of m rows and n columns. Each entry in the table is a pair of bases (in alphabetical order). We transformed this table to an $m \times n$ integer matrix A. Each entry of A is set to -1,0, or $+1$ (or is empty). Let B_1 and B_2 be the two possible alleles that appear in the j-th SNP (in alphabetical order). If the genotypic information for the j-th SNP of the i-th sample is $B_1 B_1$, then the (i, j)-th entry of A is set to $+1$; else if it is $B_1 B_2$, then the (i, j)-th entry of A is set to 0; else if it is $B_2 B_2$, then the (i, j)-th entry of A is set to -1 [4, 3].

The Singular Value Decomposition and Principal Components Analysis

We briefly describe the Singular Value Decomposition (SVD) of matrices and the related Principal Components Analysis (PCA). Given m subjects and n SNPs ($m \leq n$), let the $m \times n$ matrix A denote the subject-SNP matrix encoded as described above. After mean-centering the columns (SNP genotypes) of A, the SVD of the matrix returns m pairwise orthonormal vectors u^i, n pairwise orthonormal vectors v^i, and m non-negative singular values σ_i such that $\sigma_1 \geq \sigma_2 \geq \ldots \geq \sigma_m \geq 0$. The matrix A may be written as a sum of outer products as

$$A = \sum_{i=1}^{m} \sigma_i u^i {v^i}^T. \tag{1}$$

Each triplet (σ_i, u^i, v^i) may be used to form a principal component of A. Formally, the i-th most significant principal component of a matrix A is the rank-one matrix that is equal to $\sigma_i u^i {v^i}^T$. In our setting, the left singular vectors (the u^i's) are linear combinations of the columns (SNPs) of A and will be called eigenSNPs [2]. Notice that a principal component is a matrix, whereas an eigenSNP is just a column vector. PCA is a well-known dimensionality reduction technique that, in this case, represents all subjects with respect to a small number of eigenSNPs, corresponding to the top few principal components.

Determining the number of significant principal components

The PCAIM selection procedure that was used in our work necessitates as input the number of significant principal components. Statistical tests of significance such as the Tracy-Widom test [1] or other tests [5, 4] do not seem suitable for our goal, since, for example, when analyzing the data corresponding to the top node in Figure 1 (main text) we are only interested in assigning a sample to one of three broad groups. Towards that end, the top two principal components contain sufficient information and lower order principal components, while statistically significant, tend to differentiate cattle breeds at a finer granularity than necessary at this level. Thus, we chose to determine the significance of a principal component by measuring its contribution in separating the cattle groups that correspond to the relevant node of the decision tree. In order to achieve this, we performed the following experiment: given the data at each node of our decision tree (say m samples genotyped on n SNPs) we ran a complete leave-one-out validation experiment using our 5-NN classification algorithm and k principal components with k varying from one up to ten. Thus, we systematically leave out one of the m individuals (test set) and then we seek to predict their "ground truth" breed using the remaining $m - 1$ individuals (training set). To do that we project all m individuals on the top k eigenSNPs and identify the five Nearest Neighbors of the individual in the test set. By repeating this experiment m times (once for each of the m individuals) we compute two statistics: the classification accuracy and the average number of correctly predicted nearest neighbors (see online material for the numerical results for each value of k at each node of the decision tree). We chose to set k (the number of significant principal components) to the value that minimizes the number of misclassifications and maximizes the average number of correct nearest neighbors. In the online supporting material we have provided detailed tables with the results of the above min-max objective for each value of k from one to ten at each node of the decision tree of Figure 1 (main text). Interestingly, in most cases that were studied here, there existed a unique value of k that achieved this min-max objective for every node in Figure 1 (main text).

Table 1 in the main text (column 1) indicates the number of components that were deemed significant using the above min-max objective. An immediate observation is that the number of principal components that are significant at each node never exceeds four. In the online supporting material we provide plots of all eigenSNPs (principal components) that were deemed significant via our analysis. In these plots, it is easy to observe that in most cases the significant eigenSNPs clearly separate the targeted subsets of breeds under study at each level.

Accuracy metrics

We report two statistics. First, in a cross-validation experiment using the 5-NN classifier, we will consider a sample's breed of origin to be accurately predicted if and only if at least three out of the five nearest neighbors belong to the correct breed. The following definition is now immediate.

Definition 1 [Classification Accuracy (C_{ACC})] *The classification accuracy is defined as the percentage of the number of individuals in the test set whose breed of origin was accurately predicted.*

We should note that if an individual with breed of origin X had two nearest neighbors in X and the remaining three in – say – three distinct breeds Y_1, Y_2, and Y_3, we would consider the

individual incorrectly predicted. Thus, our accuracy metric is quite stringent, since it necessitates a strict majority. Our second metric rectifies the situation by measuring the average (over all individuals in the test set) number of nearest neighbors that belong to the correct breed of origin.

Definition 2 [Average number of correct neighbors (NN_{AVG})] *The average number of correct neighbors is defined as the average number of neighbors that belong to the correct breed of origin over all individuals in the test set.*

References

[1] S. Biswas, L. B. Scheinfeldt, and J. M. Akey. Genome-wide insights into the patterns and determinants of fine-scale population structure in humans. *Am. J. Hum. Genet.*, 84:641–650, 2009.

[2] Z. Lin and R.B. Altman. Finding haplotype tagging SNPs by use of principal components analysis. *American Journal of Human Genetics*, 75:850–861, 2004.

[3] P. Paschou, P. Drineas, J. Lewis, C. M. Nievergelt, D. A. Nickerson, J. D. Smith, P. M. Ridker, D. I. Chasman, R. M. Krauss, and E. Ziv. Tracing sub-structure in the European American population with PCA-informative markers. *PLoS Genet.*, 4:e1000114, 2008.

[4] Peristera Paschou, Elad Ziv, Esteban G Burchard, Shweta Choudhry, William Rodriguez-Cintron, Michael W Mahoney, and Petros Drineas. PCA-correlated SNPs for structure identification in worldwide human populations. *PLoS Genet*, 3(9):1672–86, 2007.

[5] N. Patterson, A.L. Price, and D. Reich. Population Structure and Eigenanalysis . *PLoS Genet*, 2(12):e190, 2006.
