# Supplementary figures and images for: Tracing Cattle Breeds with Principal Components Analysis Ancestry Informative SNPs
Source: PLoS One. 2011 Apr 7;6(4):e18007. doi: 10.1371/journal.pone.0018007 (PMC3072384; doi:10.1371/journal.pone.0018007)

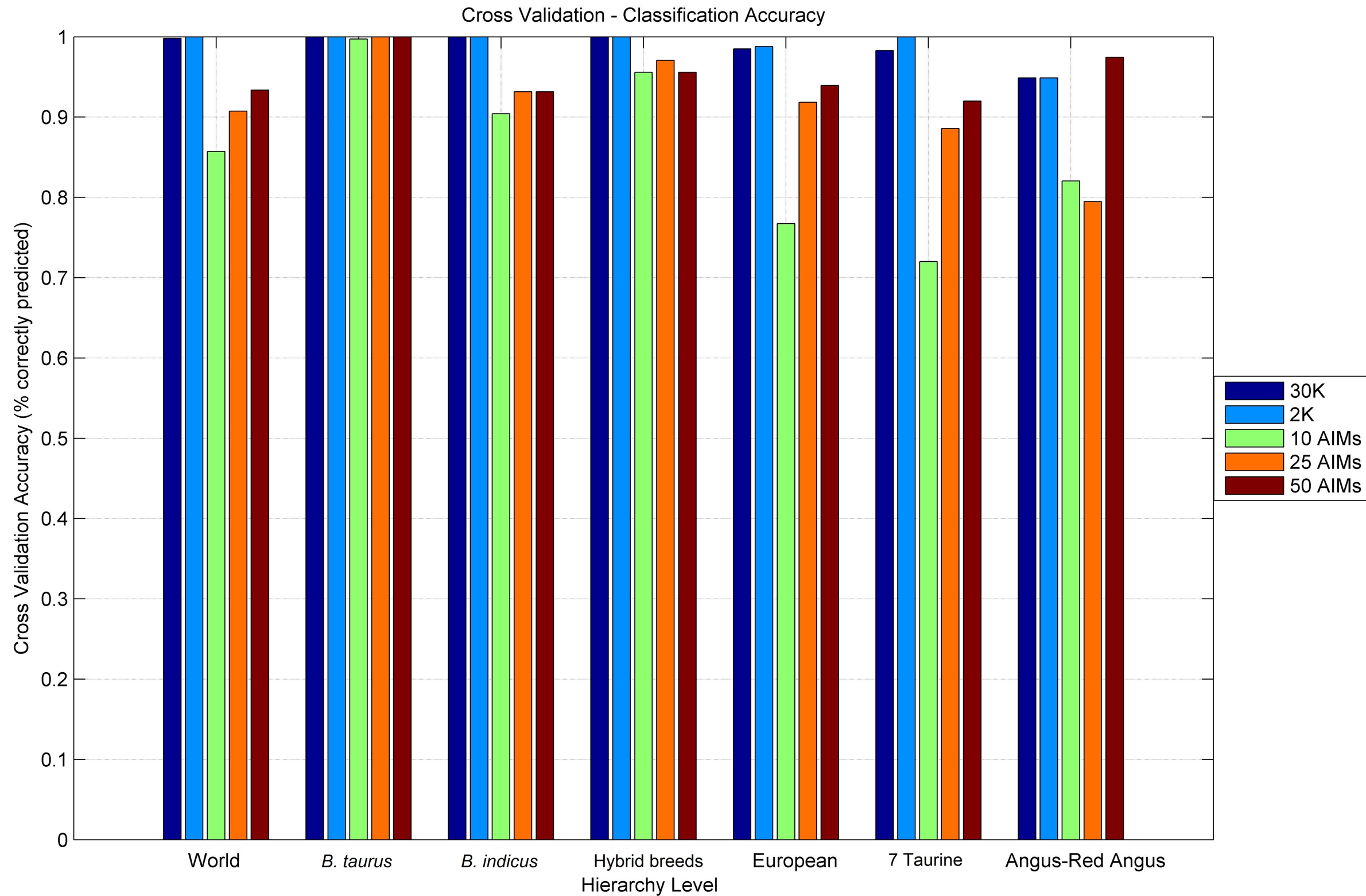

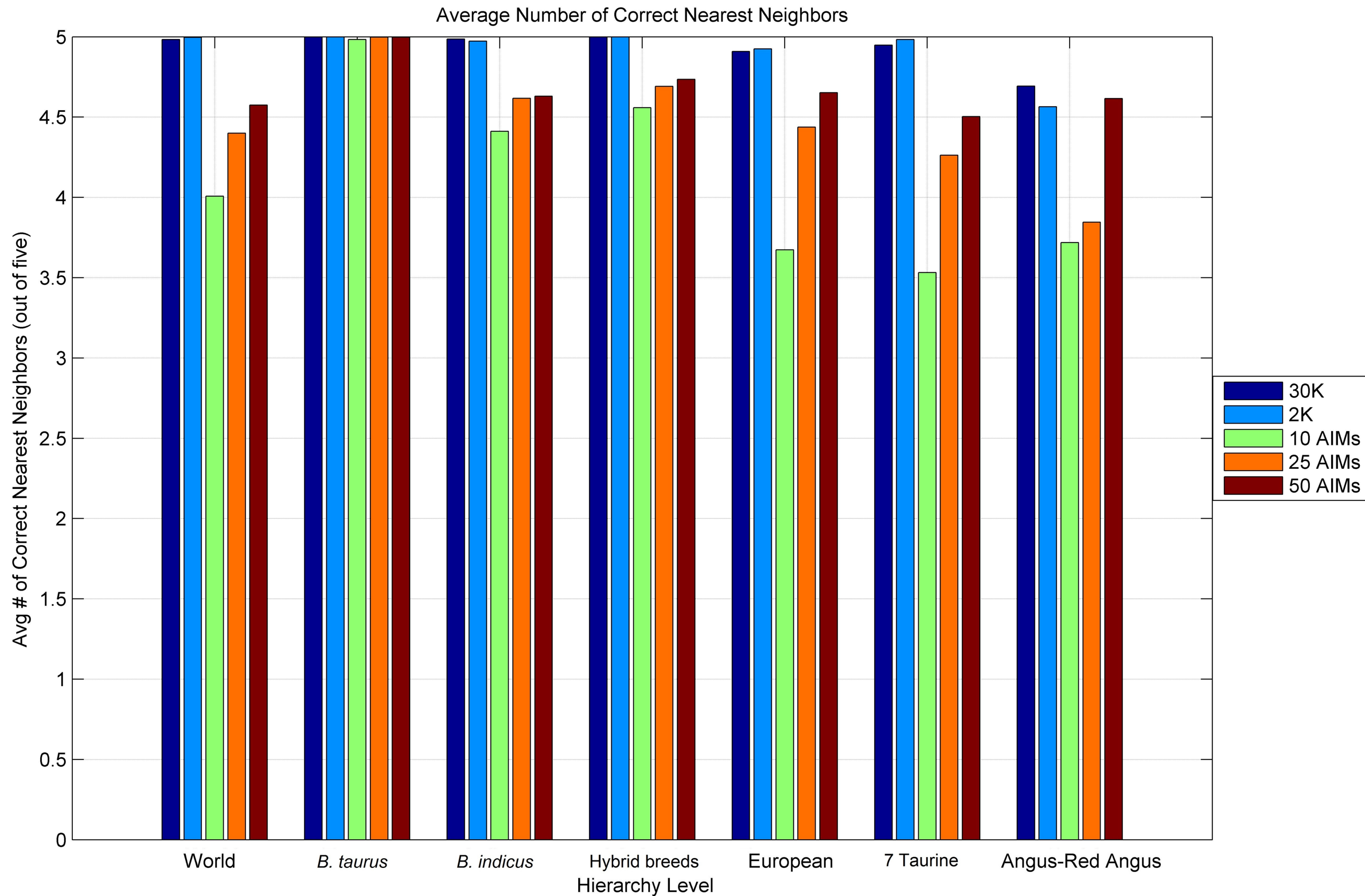

Supplement: Figure S1 — Classification accuracy with panels of sizes 10, 25, and 50 SNPs. Classification accuracy of our complete leave-one-out cross-validation experiment at all nodes of our decision tree. Five different panel sizes are evaluated, with 30K corresponding to all available markers, 2K corresponding to the top 2,000 PCAIMs, and P1, P2, and P3 corresponding to panels sizes of 10, 25, and 50 SNPs respectively at all nodes of the decision tree of Figure 1 in the main text. These smaller panels emerged by removing redundant markers from the top 2,000 AIMs. Notice that the top 2,000 markers were selected using only the individuals in the training set of the crossvalidation experiment. (A) Classification accuracy results (out of 100%). (B) Average number of correctly predicted nearest neighbors (out of five). (PDF) [file pone.0018007.s002.pdf]

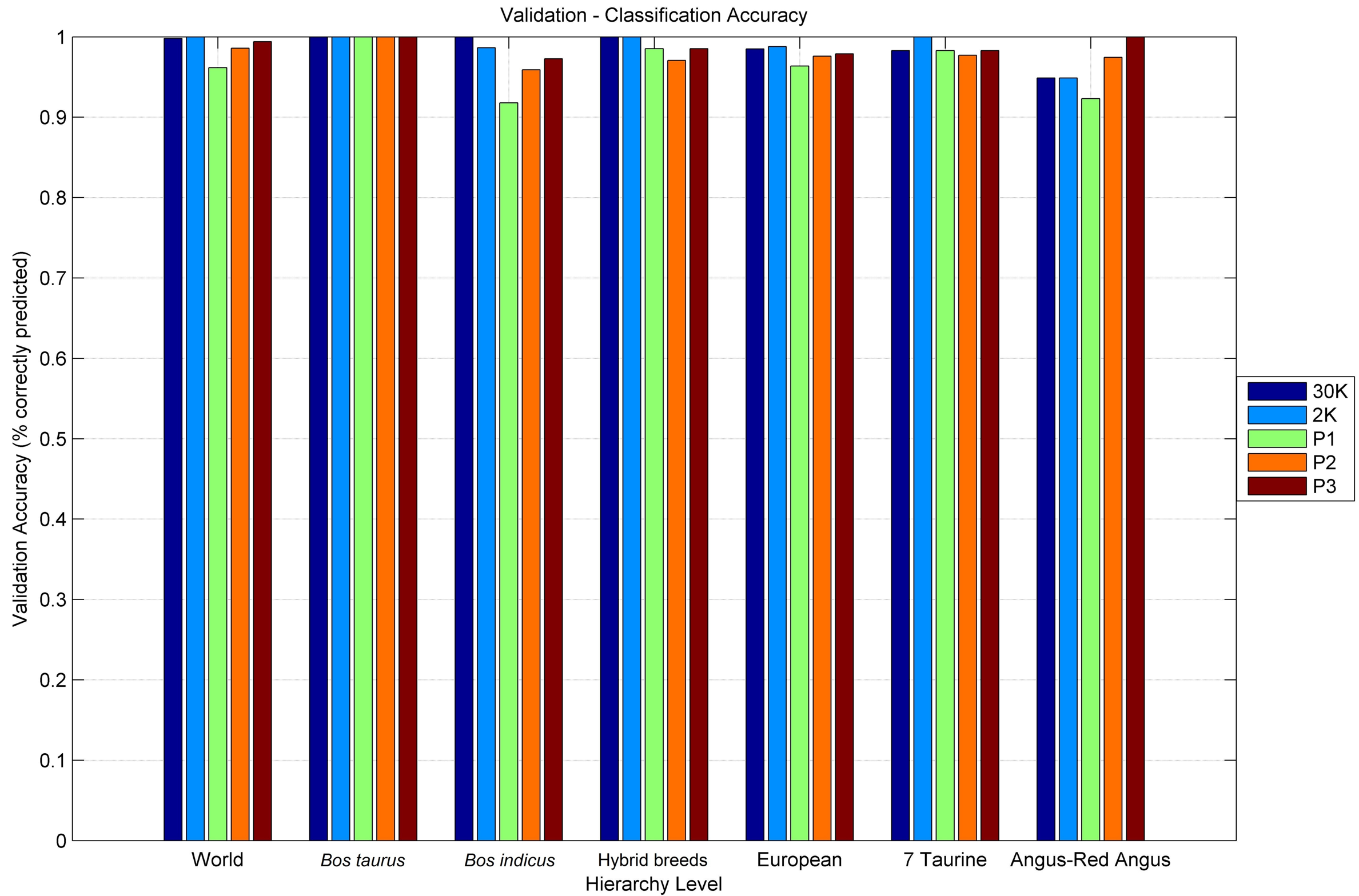

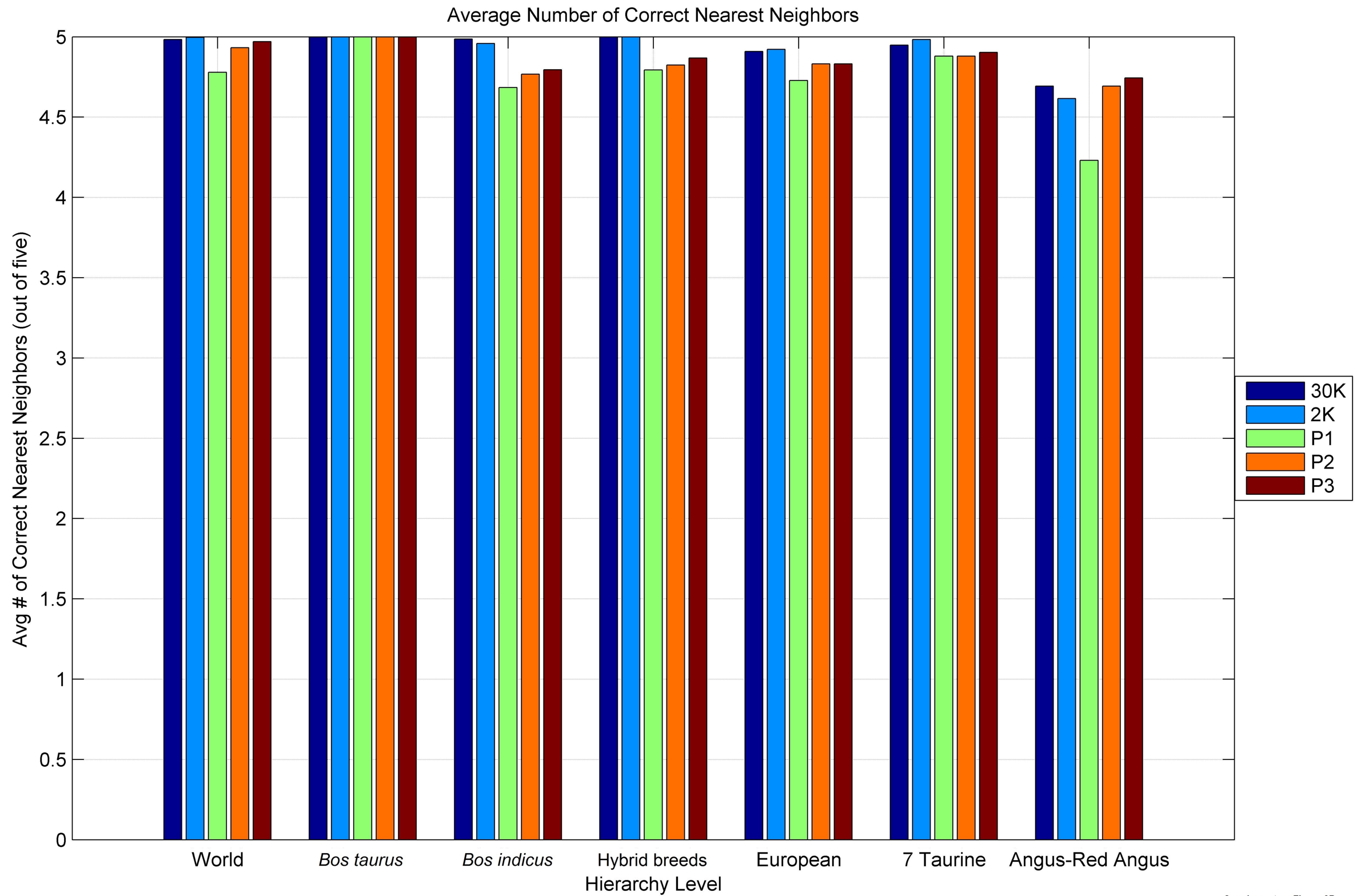

Supplement: Figure S2 — Classification accuracy of proposed panels. Classification accuracy of our proposed panels at all nodes of our decision tree. Five different panel sizes are evaluated, with 30K corresponding to all available markers, 2K corresponding to the top 2,000 PCAIMs, and P1, P2, and P3 corresponding to the panel sizes depicted in Table 1 of the main text. These smaller panels emerged by removing redundant markers from the top 2,000 AIMs. Notice that the top 2,000 markers were selected using all 497 samples, without splitting them in training and test sets, unlike the crossvalidation experiments of Figure 3 (main text). (A) Classification accuracy results (out of 100%). (B) Average number of correctly predicted nearest neighbors (out of five). (PDF) [file pone.0018007.s003.pdf]

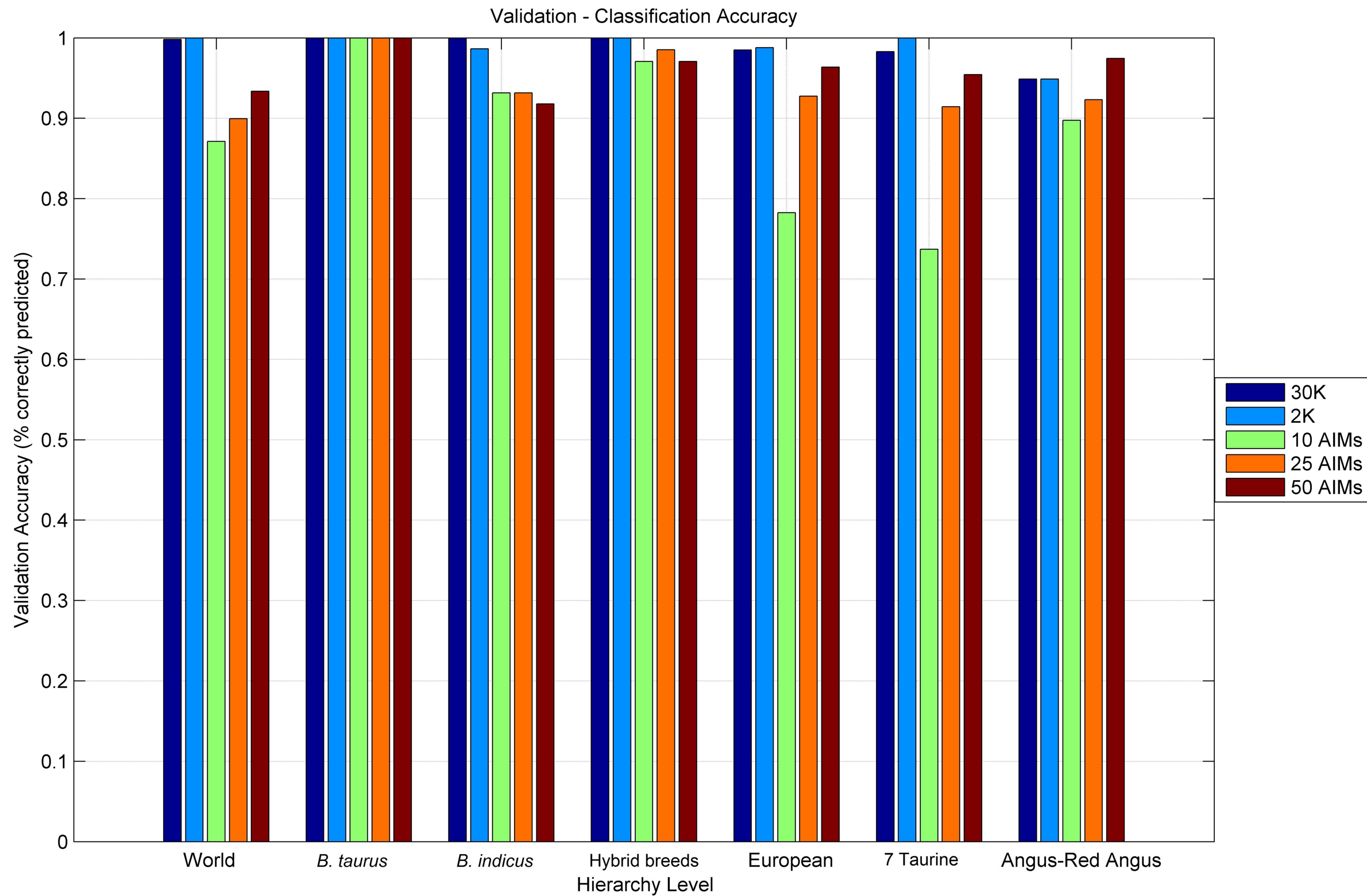

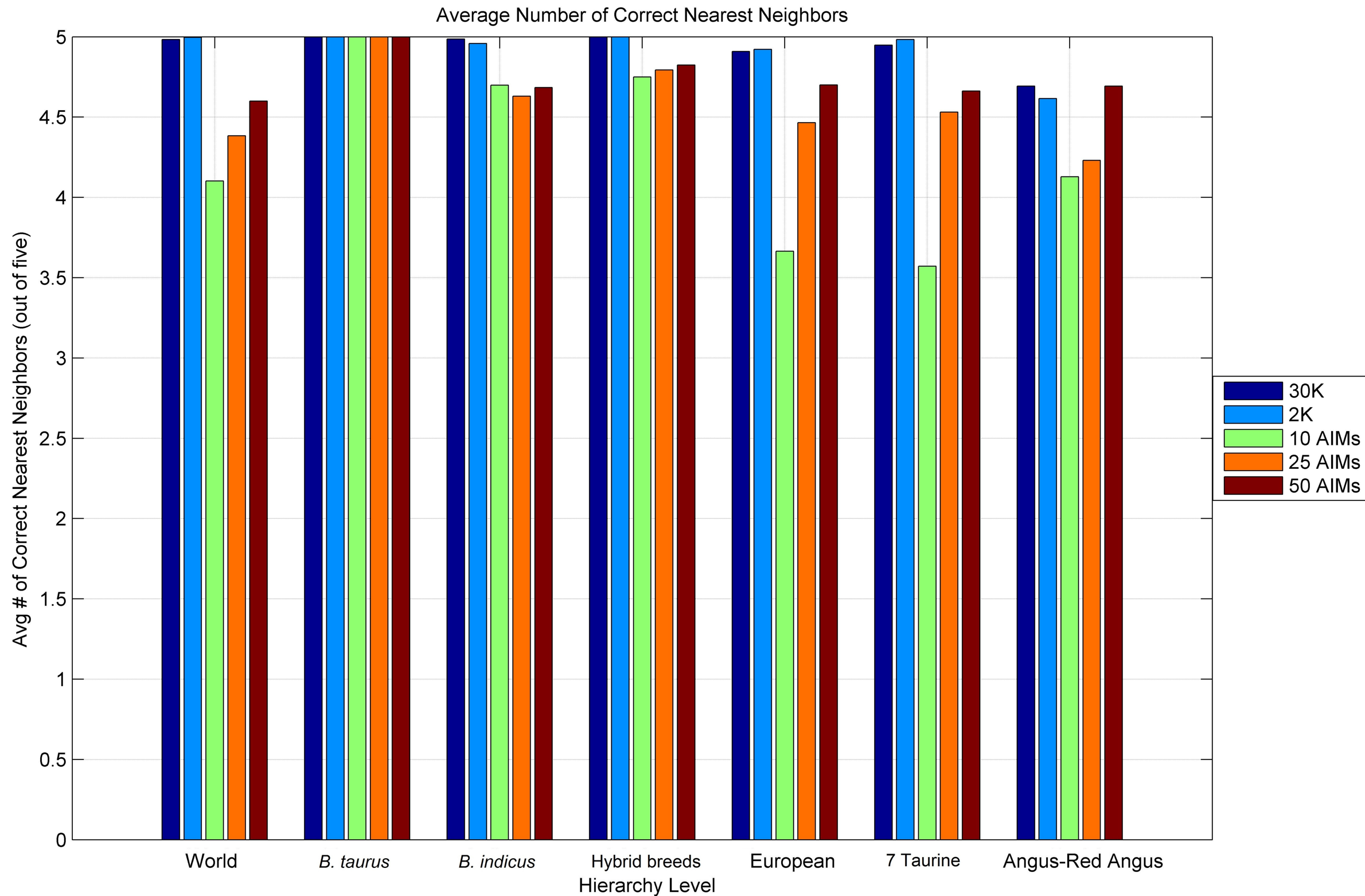

Supplement: Figure S3 — Classification accuracy of 10, 25, and 50 SNP panels. Classification accuracy of our “small” panels. Five different panel sizes are evaluated, with 30K corresponding to all available markers, 2K corresponding to the top 2,000 PCAIMs, and P1, P2, and P3 corresponding to panel sizes of 10, 25, and 50 SNPs respectively at all nodes of the decision tree of Figure 1 in the main text. These smaller panels emerged by removing redundant markers from the top 2,000 AIMs. Notice that the top 2,000 markers were selected using all 497 samples, without splitting them in training and test sets, unlike the crossvalidation experiment of Figure S1. (A) Classification accuracy results (out of 100%). (B) Average number of correctly predicted nearest neighbors (out of five). (PDF) [file pone.0018007.s004.pdf]
